# Commissioning and quality assurance of a commercial stereotactic treatment‐planning system for extracranial IMRT

**DOI:** 10.1120/jacmp.v7i1.2125

**Published:** 2006-02-21

**Authors:** Lu Wang, Jinsheng Li, Kamen Paskalev, Peter Hoban, Wei Luo, Lili Chen, Shawn McNeeley, Robert Price, Charlie Ma

**Affiliations:** ^1^ Department of Radiation Oncology Fox Chase Cancer Center Philadelphia Pennsylvania 19111 U.S.A.

**Keywords:** stereotactic IMRT, Monte Carlo, treatment‐planning system

## Abstract

A 3D treatment‐planning system (TPS) for stereotactic intensity‐modulated radiotherapy (IMRT) using a micro‐multileaf collimator has been made available by Radionics. In this work, we report our comprehensive quality assurance (QA) procedure for commissioning this TPS. First, the accuracy of stereotaxy established with a body frame was checked to ensure accurate determination of a target position within the planning system. Second, the CT‐to‐electron density conversion curve used in the TPS was fitted to our site‐specific measurement data to ensure the accuracy of dose calculation and measurement verification in a QA phantom. Using the QA phantom, the radiological path lengths were verified against known geometrical depths to ensure the accuracy of the ray‐tracing algorithm. We also checked inter‐ and intraleaf leakage/transmission for adequate jaw settings. Measurements for dose verification were performed in various head/neck and prostate IMRT treatment plans using the patient‐specific optimized fluence maps. Both ion chamber and film were used for point dose and isodose distribution verifications. To ensure that adjacent organs at risk receive dose within the expectation, we used the Monte Carlo method to calculate dose distributions and dose‐volume histograms (DVHs) for these organs at risk. The dosimetric accuracy satisfied the published acceptability criteria. The Monte Carlo calculations confirmed the measured dose distributions for target volumes. For organs located on the beam boundary or outside the beam, some differences in the DVHs were noticed. However, the plans calculated by both methods met our clinical criteria. We conclude that the accuracy of the XKnife™ RT2 treatment‐planning system is adequate for the clinical implementation of stereotactic IMRT.

PACS numbers: 87.53.Xd, 87.53.Ly, 87.53.Wz

## I. INTRODUCTION

The success of stereotactic radiosurgery in treating both benign and malignant tumors within the cranial region has logically led to attempts to mimic these treatments in extracranial regions. Stereotactic irradiation of extracranial targets has thus emerged as a new concept of treatment in clinical radiotherapy.^(^ ^1^ ^–^ ^4^ ^)^ As a response to these attempts, a commercial treatment‐planning system (TPS), XKnife™ RT2 (Radionics Inc., Burlington, MA), for stereotactic extracranial radiotherapy and intensity‐modulated radiotherapy (IMRT) has become available. It is anticipated that the development and accurate commissioning of such systems for clinical implementation will ensure the widespread use of stereotactic extracranial conformal and IMRT and facilitate dose hypofractionation/escalation for some diseases.^(^ ^5^ ^–^ ^8^ ^)^

Stereotactic irradiation of extracranial targets follows the general principles common to standard radiotherapy, but relies on stereotaxy for target localization and dose delivery, and generally requires more stringent accuracy criteria.^(9)^ The stereotaxy is established by using a stereotactic body frame (SBF) combined with a reliable immobilization system. The SBF can facilitate accurate patient positioning^(^ ^2^ ^,^ ^10^ ^,^ ^11^ ^)^ as well as precise targeting of tumors based on assigned Cartesian coordinates throughout the body. With this technique, the isocenter of a target is identified based on the coordinates rather than the patient's skin and bony landmarks. Appropriate implementation or use of this technique may result in a significant change in treatment routine compared to conventional therapy, and the technique may well facilitate CT‐based image‐guided radiotherapy.

As the target localization accuracy is improved, the demand for treatment planning accuracy of a TPS for stereotactic radiotherapy is also increased. Unlike TPSs for non‐stereotactic radiotherapy, the accuracy of a stereotactic treatment‐planning system has two components: the accuracy of stereotaxy and the accuracy of dose calculation. The first one ensures that a Cartesian coordinate system associated with a SBF is well established within the TPS for accurate determination of a target position. Both target positional accuracy and dose calculation accuracy affect dose delivery accuracy to a defined target. Therefore, commissioning a stereotactic TPS should include these two components.

The XKnife™ RT2 system is designed for both intracranial and extracranial conformal and IMRT treatment planning. Although XKnife™ RT2 has evolved from the XKnife™ system, which was designed only for stereotactic intracranial conformal radiotherapy planning and has been well accepted and in use for more than a decade, the extended application to extracranial targets makes XKnife™ RT2 different from its predecessor in the following ways: new types of fixation systems are used, and a treatment/simulation couch must be considered. In addition, IMRT adds treatment planning complexity, especially when the main application of XKnife™ RT2 has been used with a detachable micro‐multileaf collimator (Radionics Inc., Burlington, MA) on either Siemens (Siemens Medical Solutions, Concord, CA) or Varian linear accelerators (Varian Medical Systems, Inc., Milpitas, CA), whose connectivity and compatibility with the LINAC have to be rigorously ensured. In this work, we report our comprehensive procedures to commission this system for our clinical implementation of stereotactic IMRT for extracranial radiotherapy.

## II. MATERIALS AND METHODS

### A. The accuracy of stereotaxy

Stereotaxy refers to a 3D superposition of a fixed coordinate system upon a given structure (e.g., human head or body). Stereotaxy is commonly achieved with special mechanical frames, such as the SBF for extracranial applications. The frame can be rigidly attached to a baseboard to which an immobilization device is also affixed while the patient is positioned in the immobilization device for CT scanning. The accuracy of stereotaxy refers to the accuracy of the coordinate system in relation to the treatment geometry in the stereotactic planning system. One must distinguish this from the setup accuracy of a patient within the stereotactic frame because the latter refers to the reproducibility of the patient geometry in the frame. The setup accuracy of the SBF and a head/neck frame (Radionics Inc., Burlington, MA) has already been reported in the literature^(^ ^11^ ^,^ ^12^ ^)^ and will not be discussed here.

**Figure 1:**
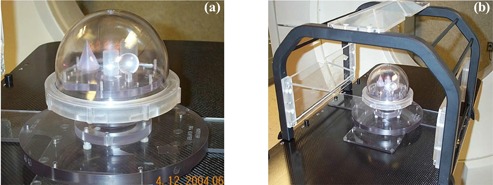
(a). The phantom used for the verification of the accuracy of stereotactic coordinate system for XKnife™ RT2. The phantom contains four geometric structures (cone, cylinder, sphere, and cube), each located at fixed positions within the phantom. (b) Setup for phantom scanning in a stereotactic body frame. The phantom is attached to a plastic plate that has three holes (upper, middle, and lower) for holding the phantom in three reproducible positions.

We used a phantom supplied by Radionics for the purpose of evaluating the accuracy of stereotaxy of the TPS. The phantom, shown in Fig. 1(a), is a plastic globe with a removable top and precisely located structures (cone, cylinder, sphere, and cube) at known positions within the phantom. The exact stereotactic coordinates for the top center of each object are known (provided by Radionics); thus we can compare them with the calculated coordinates from the planning system. Three CT scans of the phantom were acquired on a Picker PQ 5000 CT scanner (Philips Medical Systems, Cleveland, OH) with 2‐mm slice spacing, a field of view of 480 mm, and with a matrix size of 512×512 (pixel size 0.94 mm). The phantom was attached to a plastic plate that has three sets of bins and can hold the phantom in three reproducible positions (upper, middle, and lower) (see Fig. 1(b)). The plastic plate was latched onto the baseboard within the SBF while the SBF was attached to the baseboard.

After scanning, the CT images were transferred to the XKnife™ RT2 system through DICOM transfer. We contoured each of the four objects and used the Autocenter function to position the isocenter to each of the structures. Then, by moving the isocenter superiorly until it reached the top of each structure, we determined the coordinates of the top centers of these objects. The coordinates determined on the TPS were compared with the vendor‐provided values.

### B. Quality assurance of CT‐to‐electron density conversion

Although corrections for beam hardening and scattering have been implemented in currently available CT scanners, systematic differences exist between a real CT image and an ideal, artifact‐free and monochromatic image. Therefore, a well‐designed TPS should allow the user to input the site‐specific CT data measured in a phantom embedded with different materials. The corresponding ranges of electron density $\left(\bar{e}/{\text{cm}}^{3}\right)$ relative to water and the CT numbers of these materials were experimentally determined previously.^(13)^ The XKnife™ RT2 system allows site‐specific CT data to be measured for a phantom with various known electron density materials embedded. In XKnife™ RT2, the conversion of the CT number to electron density is calculated by two linear equations, represented by four fitting parameters:

$$\begin{array}{rl} & j(x)=f_{0}+f_{1}\times \text{CT},\;\text{where}\;\text{CT}<1000;\\ & k(x)=f_{2}+f_{3}\times \text{CT},\;\text{where}\;\text{CT}>1000; \end{array}$$

One curve (fitted by two parameters) is for CT numbers <1000; the other is for CT numbers >1000. Based on our site‐specific CT data for a phantom consisting of different materials with known electron densities, the fitted curves are discontinuous at a CT number of 1000. This will result in an inconsistency in the electron density values for the material with a CT number equal to 1000, depending on which curve is used. In addition, the fitting curve above the CT number of 1000 does not reproduce the correct conversion value for a polymethylmethacrylate (PMMA) or acrylic phantom used for IMRT QA. In a separate study,^(14)^ we demonstrated that whether using the site‐specific CT‐to‐electron density conversion curve or a fit for a generalized CT scanner, the patient dose distribution does not alter significantly (<2%). In contrast, for IMRT planning and QA, ensuring the accuracy of CT‐to‐electron density conversion for the QA phantom is important, since the whole phantom is made of PMMA or acrylic, with dimensions of approximately 30 cm wide, 20 cm high, and 20 cm long, and a small variation in electron density can introduce a significant change in the equivalent path length calculated for large depths. Therefore, we have readjusted the fitting parameters to ensure the continuity and uniqueness of the conversion curve, as well as the conversion accuracy for the PMMA phantom. For the acrylic phantom, relative electron density is a factor 1.14 larger than that of water. This factor is smaller than the relative *physical* density of 1.17. Since it is the electron density that determines radiological path length, this parameter should be correctly reflected in the CT‐to‐electron density conversion. Since the most probable CT number for our PMMA phantom is 1200, this CT number has been assigned to have a relative electron density of 1.14. The fitting parameters were adjusted accordingly based on this data point.

### C. Quality assurance of leaf transmission and interleaf leakage of the micromultileaf collimator

**Figure 2:**
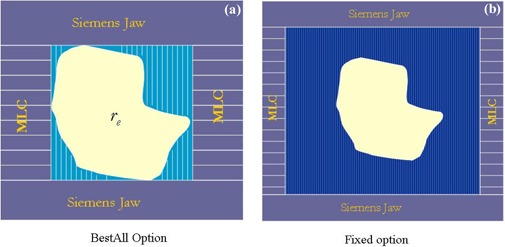
LINAC jaw settings when the attached mMLC is used for IMRT delivery. Figure 2(a) shows the BestAll option, which sets the LINAC jaws to encompass each of the mMLC fields (per gantry angle). Figure 2(b) shows the Fixed option, which sets a fixed jaw position (e.g., $10\times 10\;{\text{cm}}^{2}$) for all the mMLC fields (per gantry angle).

One of the advantages of XKnife™ RT2 is that it can perform treatment planning for intensity modulation based not only on commonly available multileaf collimators (MLCs) (e.g., Siemens MLC and Varian Millennium 120 MLC), but also by modeling a micro‐MLC (mMLC) (Radionics Inc., Burlington, MA) with a leaf width of 4.0 mm at the isocenter. Our commissioning has been for this detachable mMLC on a Siemens Primart machine. When IMRT plans are delivered by the mMLC, there are two options for setting up the Siemens jaws: one is BestAll, and the other is Fixed. As shown in Fig. 2, the BestAll option sets the Siemens jaws to encompass each of the mMLC fields (per gantry angle), while the Fixed option sets a fixed jaw position (e.g., $10\times 10\;{\text{cm}}^{2}$) for all the mMLC fields. It is obvious that from the radiation leakage point of view, the BestAll option is superior. On the other hand, the Fixed option may present technical simplicity for treatment delivery when the leaf leakage is negligible.

To ensure that the leaf leakage is acceptable, we measured the mMLC transmission and interleaf leakage by ion chamber and film. The chamber measurements were performed at the calibration conditions (source‐to‐surface (SSD)=100cm, field size $10\times 10\;{\text{cm}}^{2}$ at $d_{\max}$) for both open and closed mMLC exposure. The chamber orientation was perpendicular to the leaf motion direction and was about 2 cm away from the central line of leaf closure. The film measurement was scanned by film dosimetry software (RIT113 V4) to measure the inter‐ and intraleaf leakage.

### D. Quality assurance of dose calculation by measurements

Before conducting any clinical measurements, we checked the dose calculation algorithm, in particular, the ray‐tracing algorithm in the planning mode. Since XKnife™ RT2 is based on XKnife™, which was designed for stereotactic intracranial treatment planning, it is important to verify that the ray‐tracing algorithm has been correctly modified to deal with the CT couch, a device that will not appear in the intracranial situation but will be involved in the CT scans for extracranial applications. We scanned our QA phantom made of PMMA and checked the radiological path lengths for beams that pass through the couch. Based on the known geometrical depths and the electron density of the phantom, we could check the accuracy of the ray‐tracing algorithm when a CT couch is involved. Our work revealed a limitation of the previous version of the TPS, which incorrectly accounts for the CT couch in the dose calculation. Thus, we notified the vendor and had this error corrected in the new release. The results reported here were all based on the new version (XKnife™ RT v.2.1.2) using fixed jaw options.

Film measurements were performed to ensure that the measured fluence maps agreed with the planned maps. We then conducted chamber measurements in the QA phantom for 10 test cases to verify point doses. These 10 cases included IMRT plans for prostate cancer, lung tumor, brain tumor, and head and neck tumor. Treatment planning included both coplanar and noncoplanar beam arrangements. Isodose distributions were measured using the Kodak EDR film. The exposed film was scanned into the RIT film dosimetry system (Radiological Imaging Technology Inc., Colorado Springs, CO, v.4). The XKnife™ RT2 calculated isodose distributions were also imported to the RIT system. Comparisons between the calculated and the measured dose distributions were made within the RIT system.

### E. Quality assurance of DVHs by the Monte Carlo method

In XKnife™ RT2, a measurement‐based pencil beam algorithm is used^(15)^ for forward dose calculation, and a fast tissue maximum ratio (TMR) method is used for inverse optimization dose calculation. The pencil beam kernel includes primary and limited scatter dose with a predefined Gaussian spread. The limitation of not separating the scatter from the primary dose should not cause significant problems in a homogeneous medium. However, the limitation that only the fast TMR method can be used for IMRT planning could be a problem for modulated fields if neighboring beam elements are taken into account in an approximate manner during implementation using the Monte Carlo kernels. In addition, the inhomogeneity correction in XKnife™ RT2 is based on equivalent path length calculations along the primary beam direction. To test the degree of the inaccuracy due to these approximations, we used the Monte Carlo method in our comprehensive commissioning process. We have developed several EGS4‐based user codes in our group for both beam simulation and dose calculation for direct in‐patient Monte Carlo dose calculation.^(^ ^16^ ^,^ ^17^ ^)^ These source codes were all validated by measurements.^(^ ^18^ ^,^ ^19^ ^)^ In the Monte Carlo dose calculation, the average leakage resulting from intra‐ and interleaf leakages is considered. The tongue‐and‐groove effect is not included. However, Deng et al.^(20)^ demonstrated this effect to be small (<1.6%).

One limitation with the current version of XKnife™ RT2 is that it does not allow for RTOG Export/Import. Therefore, the contours of tumor target and organs at risk cannot be used directly in our Monte Carlo dose calculation. To get around this limitation, contours were drawn by a physician on the AcQsim (Philips Medical Systems, Cleveland, OH) treatment‐planning system instead of on XKnife™ RT2. The same contours were then exported to the Monte Carlo dose calculation program and to XKnife™ RT2, for which a small program was written to compile the contour files with the format that XKnife™ RT2 accepts.

We performed the Monte Carlo dose calculation for nine prostate. IMRT plans using the patient‐specific CT data, the same beam configurations, and the same intensity maps generated in XKnife™ RT2. Based on the patient‐specific CT data, one can define the computational geometry and electron density information. The intensity maps were built based on the leaf sequence files generated by XKnife™ RT2. The effects of photon scattering and leakage were considered in this process. The photon beam was represented using a three‐source model, and it was commissioned using a standard set of measured data.

Monte Carlo dose calculations for these IMRT plans were compared with those generated by XKnife™ RT2. We specifically compared $D_{95}$ and $D_{05}$ for the target (the doses received by the 95% and 5% of the target volume). We also compared $V_{40}$ and $V_{65}$ for the rectum and bladder to determine whether there are significant differences in the beam penumbra regions that will affect the doses to critical structures.

## III. RESULTS

### A. The accuracy of stereotaxy

The coordinates of the top centers of each of the four objects determined by the TPS (XKnife™ RT2) are shown in the Table 1. The known phantom specifications are also listed in the table. The differences between the two are presented in the last three columns. The study was performed at three different locations, and the table includes data comparisons for the three positions. The average differences and standard deviations of the differences, which reflect the error bars in determining these coordinates, were derived by summing all the differences in the last three columns. It is found that the accuracy of the stereotaxy as determined by the treatment‐planning system is 0.63±0.5mm,0.57±0.28mm, and 0.58±0.46mm in A‐P, lateral, and vertical directions, respectively, when using a slice thickness of 2 mm. An increase in slice thickness may affect the accuracy in the inferior‐superior (vertical) direction.

**Table 1:** Comparison of coordinates of the top centers of each object determined in the TPS with the known specification at three different positions

Position 1									
	Phantom specs (mm)			XKnife RT (mm)			Difference (mm)		
Object	AP	Lat	Vert	AP	Lat	Vert	AP	Lat	Vert
cylinder	14.0	0.0	100.0	14.6	0.1	101.1	0.6	0.1	1.1
cube	24.0	17.0	120.0	24.4	17.6	121.1	0.4	0.6	1.1
cone	24.0	20.0	65.0	23.4	20.4	64.9	0.6	0.4	0.1
sphere	16.7	−20.0	125.0	17.9	−19.4	125.1	1.2	0.6	0.1
Average							0.7	0.4	0.6
Position 2									
	Phantom specs (mm)			XKnife RT (mm)			Difference (mm)		
Object	AP	Lat	Vert	AP	Lat	Vert	AP	Lat	Vert
cylinder	14.0	0.0	−0.6	14.5	0.2	−0.8	0.5	0.2	0.2
cube	24.0	17.0	19.4	24.5	16.4	18.9	0.5	0.6	0.5
cone	24.0	20.0	−35.6	22.9	19.0	−34.7	1.1	1.0	0.9
sphere	16.7	−20.0	24.4	17.0	−20.3	25.2	0.3	0.3	0.8
Average							0.6	0.5	0.6
Position 3									
	Phantom specs (mm)			XKnife RT (mm)			Difference (mm)		
Object	AP	Lat	Vert	AP	Lat	Vert	AP	Lat	Vert
cylinder	14.0	0.0	−100.6	14.3	−0.7	−100.6	0.3	0.7	0.0
cube	24.0	17.0	−80.6	24.1	16.3	−80.5	0.1	0.7	0.1
cone	24.0	20.0	−135.6	22.2	19.0	−134.6	1.8	1.0	1.0
sphere	16.7	−20.0	−75.6	16.8	−20.6	−74.5	0.1	0.6	1.1
Average							0.6	0.8	0.6

### B. CT‐to‐electron density conversion

**Figure 3:**
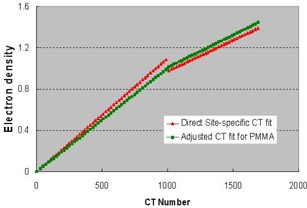
The directly fitted CT‐to‐electron density conversion curve using the site‐specific CT scanning data for a density phantom (red line). The curve was modified to ensure the CT‐to‐electron density conversion accuracy for the PMMA phantom.

Figure 3 compares the fitted CT‐to‐electron density curve and that adjusted using the relative electron density of the PMMA phantom. Although the second curve deviates from the first one, the effect on patient dose calculation is negligible.^(14)^ Nevertheless, the accuracy of dose calculation in the PMMA phantom (our QA phantom) and the agreement with the measured dose were greatly improved, depending on the depth used. For example, for a geometrical depth of 10 cm, the correct radiological path length in acrylic phantom should be about 11.3 cm, or about 1 cm larger than that calculated using the direct fitting curve (which gives a CT‐to‐electron density ratio of 1.096). For a 6‐MV photon beam, a difference of 1 cm results in a difference in the depth dose of approximately 3% to 4%.

### C. Transmission and interleaf leakage

**Figure 4:**
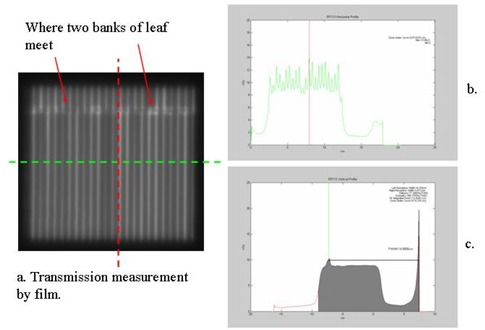
(a) The image of film exposed to 1000 MU when all the mMLC leaves were closed; (b) the transmission profile along the green line, which is perpendicular to the leaf motion; (c) the transmission profile along the red line, which is parallel to the leaf motion.

Figure 4(a) shows the image of the film exposed to 1000 MU when all the leaves were closed. Figures 4(b) and 4(c) show the transmission profiles along the two major directions: one is perpendicular to the leaf motion (along the green line in Fig. 4(a)), and the other is parallel to the leaf motion (along the red line). Figure 4(b) shows that the intraleaf leakage is about 14 cGy for a 1000 cGy delivery, and the interleaf leakage is about 8.5 cGy for 1000 cGy. Therefore, the intraleaf leakage is about 1.4%, while the transmission under the leaf is about 0.85%. The average transmission measured by a Farmer chamber of 2.7 cm length and 0.6 cm in diameter is 0.89%. The transmission at the position where the two banks of leaves meet (or intrabank leakage) is 1.5% (see Fig. 4(c)).

### D. Quality assurance of dose calculation by measurements

#### D.1 Output factor

Because of the low transmission of the mMLC, we decided to use the Fixed option for its technical simplicity. The dose algorithm implemented in XKnife™ RT2 only accounts for the effective field size created by the beam segment formed by the mMLC. Thus, the unaccounted for increase in output due to the wider fixed jaws could result in a higher measured dose compared to the calculation. To overcome this problem, we used a scaling factor to apply to the output factor to reduce the monitor units (and dose) for the fixed open jaws. Table 2 shows the comparison between the calculated and the measured doses for 10 IMRT plans using a scaling factor of 1.04 (e.g., increase the output factor by 4% in order to reduce the MUs by approximately 4%). The average difference for all 10 cases is +0.4%. This implies that, in general, this scaling factor has always made the measured dose equal to the calculated dose.

**Table 2:** Comparison of isocenter doses between the calculated and the measured doses for 10 IMRT plans

Patient	Calculated (Gy)	Measured (Gy)	Difference (%)
1^a^	2.151	2.136	−0.72%
2	1.485	1.475	−0.68%
3	2.141	2.170	1.33%
4^b^	1.485	1.520	2.30%
5^c^	1.605	1.637	1.96%
6	2.232	2.265	1.45%
7	2.045	2.065	0.96%
8	2.035	2.046	0.53%
9 (lung)	1.539	1.526	−0.87%
10	1.636	1.600	−2.26%

^a^ Prostate patient. Isodose distributions are shown in this paper.

^b^ Brain tumor patient. Isodose distributions are shown here.

^c^ Head/neck tumor patient. Noncoplanar beams were used, and isodose distributions are shown here.

#### D.2 Fluence map

**Figure 5:**
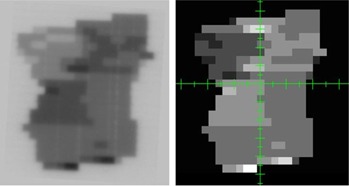
Comparison of a calculated intensity map (right) with the measured fluence map (left) for a head/neck patient. Note that high intensity (lighter pixels in the intensity map) corresponds to darker exposure on the film.

The calculated intensity maps were checked against the measured fluence maps for a head/neck patient. Such comparisons are used mainly to check the geometrical accuracy of the mMLC leaf movement. The intensity map does not include the effect of the extended source and leakage and scatter from the mMLC leaves. Figure 5 shows both images to demonstrate the agreement.

#### D.3 Isodose distributions

**Figure 6:**
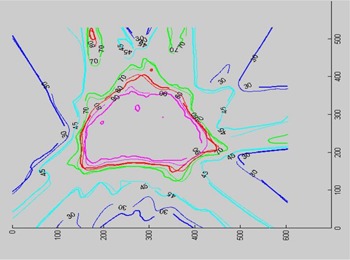
Isodose distribution comparison for a prostate IMRT plan. Isodose lines of 90%, 80%, 70%, 50%, and 30% are shown.

**Figure 7:**
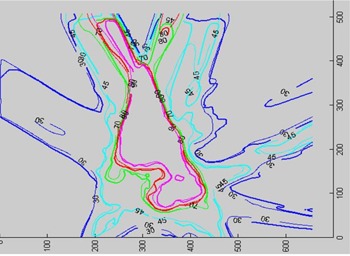
Isodose distribution comparison for a brain IMRT plan. Isodose lines of 90%, 80%, 70%, 45%, and 30% are shown.

**Figure 8:**
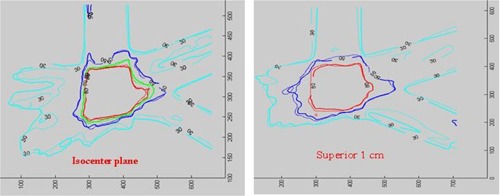
Isodose distribution comparison for a head/neck noncoplanar IMRT plan. Isodose lines of 80%, 70%, 50%, and 30% are shown in the isocenter plane (left), while only 80%, 50%, and 30% isodose lines are shown in the plane 1 cm superior to the isocenter (right).

Film measurement was performed for several IMRT plans. Here, we show distribution comparisons for three cases: (1) a prostate patient using six coplanar beams; (2) a brain tumor patient using five coplanar beams; and (3) a head/neck tumor patient with five noncoplanar beams. In Figs. 6 to 8, the measured distributions are represented by thick lines, and the calculated distributions are shown by thin lines. Figure 6 compares the dose distributions with 90%, 80%, 70%, 50%, and 30% isodose lines for a prostate IMRT plan. Figure 7 shows the dose distributions for a brain IMRT plan. Figure 8 displays the isodose distribution comparisons for a head/neck noncoplanar IMRT plan. For noncoplanar beams, the isodose accuracy was checked in the plane 1 cm superior to the isocenter plane From these figures it is seen that the agreement between the calculated and the measured dose is within 2% in the high‐dose regions for all cases.

### E. Monte Carlo verification of DVHs

**Figure 9:**
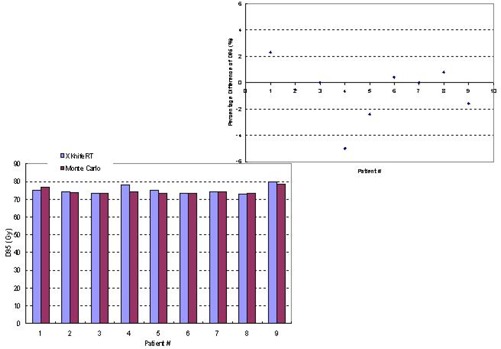
Comparison between Monte Carlo dose calculations and XKnife™ RT2 treatment planning for $D_{95}$ of the planning tumor volume for nine patients. The percentage differences between the two methods are shown in the upper figure.

**Figure 10:**
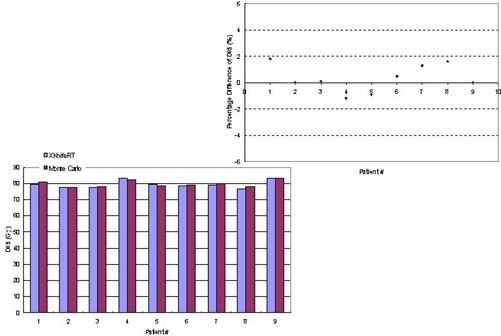
Comparison between Monte Carlo dose calculations and XKnife™ RT2 treatment planning for $D_{05}$ of the planning tumor volume for nine patients. The percentage differences between the two methods are shown in the upper figure.

Figures 9 and 10 compare $D_{95}$ and $D_{05}$ of the planning tumor volume, respectively, in histograms between Monte Carlo dose calculations and XKnife™ RT2 treatment planning. The differences between the two methods are shown in the upper figures. It is seen that, except for one case, the differences in $D_{95}$ and $D_{05}$ are within 2.5%.

**Figure 11:**
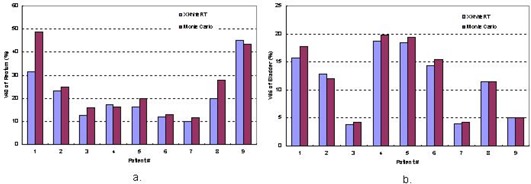
Comparison between Monte Carlo dose calculations and XKnife™ RT2 treatment planning for $V_{40}$ of the rectum and $V_{65}$ of the bladder for nine patients.

We also compared $V_{40}$ for the rectum in Fig. 11(a) and $V_{65}$ for the bladder in Fig. 11(b) for all nine patients because these parameters are checked clinically for plan acceptance. It is seen that except for patient 1, who presented a 17% difference in $V_{40}$, the rest of the patients showed a maximum difference of 3.8% in $V_{40}$ (the volume receiving 40 Gy) of the rectum for the dose prescription of 74 Gy in 37 fractions. For all patients, the average difference is 3.75% in $V_{40}$. The differences in $V_{65}$ of the bladder are even smaller, with a maximum difference of 2.1% and an average difference of 0.56%.

## IV. DISCUSSION AND CONCLUSION

The accuracy of the Radionics image fusion package (v.2) has been assessed and reported by other investigators.^(21)^ In this work, we focused mainly on commissioning of IMRT functionality of XKnife™ RT2. To commission this system for IMRT delivery using a mMLC, we took a few extra steps for the quality assurance of the system. Our first step was to check the accuracy of stereotaxy that is established with a SBF within the TPS to ensure accurate determination of target position. For accurate IMRT dose verification by measurement, the fitting parameters of the CT‐to‐electron density curve were not only rederived from the site‐specific CT scan data but also readjusted to correctly convert the CT number of the IMRT QA phantom to the electron density.

As mentioned above, our commissioning procedure is for a detachable mMLC on a Siemens Primart accelerator. The use of an add‐on mMLC for IMRT delivery may create incompatible problems because treatment delivery is not controlled only by a record‐and‐verify system but by a second system, called the mMLC controller. Another issue is the Siemens jaw (and MLC) settings. Although the BestAll option may reduce radiation leakage by setting the Siemens jaws to encompass each of the maximum mMLC fields (per gantry angle), the current version has resulted in an incompatibility problem in which the Siemens jaw setting is inconsistent with or exceeds the maximum mMLC setting when the mMLC travels to its maximum limit. In order to use the Fixed option, we measured inter‐ and intraleaf transmission (or leakage). The measurement results show that the Fixed option is adequate for stereotactic IMRT treatment planning.

Based on the measurement data presented in this paper, the dosimetric accuracy has met the published acceptance criteria.^(^ ^22^ ^–^ ^24^ ^)^ Our Monte Carlo dose verification has provided additional quality assurance. For the planning target volumes, the Monte Carlo dose calculation agrees well with the treatment planning, in terms of $D_{95}$ and $D_{05}$. For the rectum, the two methods presented an average difference of 3.8% for the percentage volume of the rectum receiving 40 Gy. Part of the difference may result from the pixel compression (from 512×512 to 128×128 pixels) for the Monte Carlo dose calculation, which could affect contour definition and, thus, DVHs. This may also be attributed to the fact that parts of the critical structures are located in the dose falloff regions; thus any inaccuracy in dose calculation and modeling of scatter variations due to the changes of depth and medium density could result in significant changes in the values of DVHs for the adjacent critical structures. Judging from our clinical criteria, the DVH of the rectum and the $V^{40}$ parameter predicted by both methods are all acceptable for treatment execution. In fact, given that the dose is delivered in 37 fractions, and daily setup uncertainty may be a bigger contribution to changes in DVHs of the adjacent structures, the above differences may not be clinically significant. From this point of view, the influence on DVHs caused by dosimetric differences should not be clinically significant. The difference in the DVH of the bladder is generally very small, with an average of 0.56% for the percentage volume of the bladder receiving 65 Gy when the total dose prescription is 74 Gy in 37 fractions.

In summary, the accuracy of XKnife™ RT2 is acceptable for stereotactic IMRT treatment planning, and it has been used clinically at the Fox Chase Cancer Center since 2004.

## References

[1] Lax I , Blomgren H , Naslund I , Svanstrom R . Stereotactic radiotherapy of malignancies in the abdomen. Methodological aspects. Acta Oncol. 1994;33:677–683. 7946448 10.3109/02841869409121782

[2] Lohr F , Debus J , Frank C , et al. Noninvasive patient fixation for extracranial stereotactic radiotherapy. Int J Radiat Oncol Biol Phys. 1999;45:521–527. 10487580 10.1016/s0360-3016(99)00190-x

[3] Wulf J , Hadinger U , Oppitz U , Olshausen B , Flentje M . Stereotactic radiotherapy of extracranial targets: CT‐simulation and accuracy of treatment in the stereotactic body frame. Radiother Oncol. 2000;57:225–236. 11054527 10.1016/s0167-8140(00)00226-7

[4] Shiu AS , Chang EL , Ye JS , et al. Near simultaneous computed tomography image‐guided stereotactic spinal radiotherapy: An emerging paradigm for achieving true stereotaxy. Int J Radiat Oncol Biol Phys. 2003;57:605–613. 14529763 10.1016/s0360-3016(03)00792-2

[5] Hof H , Herfarth KK , Munter M , et al. Stereotactic single‐dose radiotherapy of stage I non‐small‐cell lung cancer (NSCLC). Int J Radiat Oncol Biol Phys. 2003;56:335–341. 12738306 10.1016/s0360-3016(02)04504-2

[6] Timmerman R , Papiez L , McGarry R , et al. Extracranial stereotactic radioablation: Results of a phase I study in medically inoperable stage I non‐small cell lung cancer. Chest 2003;124:1946–1955. 14605072 10.1378/chest.124.5.1946

[7] Gunven P , Blomgren H , Lax I . Radiosurgery for recurring liver metastases after hepatectomy. Hepatogastroenterology 2003;50:1201–1204. 14571698

[8] Herfarth KK , Debus J , Lohr F , et al. Stereotactic single‐dose radiation therapy of liver tumors: Results of a phase I/II trial. J Clin Oncol. 2001;19:164–170. 11134209 10.1200/JCO.2001.19.1.164

[9] Podgorsak EB , Podgorsak MB . The modern technology of radiation oncology. Madison (WI): Medical Physics Publishing; 1999.

[10] Herfarth KK , Debus J , Lohr F , et al. Extracranial stereotactic radiation therapy: Setup accuracy of patients treated for liver metastases. Int J Radiat Oncol Biol Phys. 2000;46:329–335. 10661339 10.1016/s0360-3016(99)00413-7

[11] Wang L , Rojymon J , Chen L , Feigenberg S , Konski A , Ma CM . Stereotactic IMRT for prostate cancer: Setup accuracy of a new stereotactic body localization system. J Appl Clin Med Phys. 2004;5:18–28. 15738910 10.1120/jacmp.v5i2.1947 PMC5723461

[12] Fairclough‐Tompa L , Larsen T , Jaywant SM . Immobilization in stereotactic radiotherapy: The head and neck localizer frame. Med Dosim. 2001;26:267–273. 11704463 10.1016/s0958-3947(01)00074-7

[13] Matsufuji N , Tomura H , Futami Y , et al. Relationship between CT number and electron density, scatter angle and nuclear reaction for hadron‐therapy treatment planning. Phys Med Biol. 1998;43:3261–3275. 9832015 10.1088/0031-9155/43/11/007

[14] Mora G , Li G , Wang L , Ding M , Yang J , Ma C . Effect of CT conversion on Monte Carlo dose calculations for head and neck treatments. Med Phys. 2003;30:1452.

[15] Bortfeld T , Schlegel W , Rhein B . Decomposition of pencil beam kernels for fast dose calculations in three‐dimensional treatment planning. Med Phys. 1993;20:311–318. 8497215 10.1118/1.597070

[16] Wang L , Chui CS , Lovelock M . A patient‐specific Monte Carlo dose‐calculation method for photon beams. Med Phys. 1998;25:867–878. 9650174 10.1118/1.598262

[17] Ma CM , Li JS , Pawlicki T , et al. A Monte Carlo dose calculation tool for radiotherapy treatment planning. Phys Med Biol. 2002;47:1671–1689. 12069086 10.1088/0031-9155/47/10/305

[18] Wang L , Lovelock M , Chui CS . Experimental verification of a CT‐based Monte Carlo dose‐calculation method in heterogeneous phantoms. Med Phys. 1999;26:2626–2634. 10619248 10.1118/1.598802

[19] Li JS , Pawlicki T , Deng J , Jiang SB , Mok E , Ma CM . Validation of a Monte Carlo dose calculation tool for radiotherapy treatment planning. Phys Med Biol. 2000;45:2969–2985. 11049183 10.1088/0031-9155/45/10/316

[20] Deng J , Pawlicki T , Chen Y , Li J , Jiang SB , Ma CM . The MLC tongue‐and‐groove effect on IMRT dose distributions. Phys Med Biol. 2001;46:1039–1060. 11324950 10.1088/0031-9155/46/4/310

[21] Parker BC , Shiu AS , Maor MH , et al. PTV margin determination in conformal SRT of intracranial lesions. J Appl Clin Med Phys. 2002;3:176–189. 12132939 10.1120/jacmp.v3i3.2561 PMC5724599

[22] Fraass B , Doppke K , Hunt M , et al. American Association of Physicists in Medicine Radiation Therapy Committee Task Group 53: Quality assurance for clinical radiotherapy treatment planning. Med Phys. 1998;25:1773–1829. 9800687 10.1118/1.598373

[23] Van Dyk J , Barnett RB , Cygler JE , Shragge PC . Commissioning and quality assurance of treatment planning computers. Int J Radiat Oncol Biol Phys. 1993;26:261–273. 8491684 10.1016/0360-3016(93)90206-b

[24] Venselaar J , Welleweerd H , Mijnheer B . Tolerances for the accuracy of photon beam dose calculations of treatment planning systems. Radiother Oncol. 2001;60:191–201. 11439214 10.1016/s0167-8140(01)00377-2

